# Toxicological Effects of Traumatic Acid and Selected Herbicides on Human Breast Cancer Cells: In Vitro Cytotoxicity Assessment of Analyzed Compounds

**DOI:** 10.3390/molecules24091710

**Published:** 2019-05-02

**Authors:** Agata Jabłońska-Trypuć, Urszula Wydro, Elżbieta Wołejko, Andrzej Butarewicz

**Affiliations:** Department of Chemistry, Biology and Biotechnology, Faculty of Civil and Environmental Engineering, Bialystok University of Technology, Białystok, Poland, Wiejska 45E Street, 15-351 Białystok, Poland; u.wydro@pb.edu.pl (U.W.); e.wolejko@pb.edu.pl (E.W.); a.butarewicz@pb.edu.pl (A.B.)

**Keywords:** herbicide, breast cancer, mixture toxicity, cytotoxicity, traumatic acid

## Abstract

The main consequence of herbicides use is the presence of their residues in food of plant origin. A growing body of evidence indicates that herbicides cause detrimental effects upon human health while demonstrating a direct link of pesticides exposure with the occurrence of human chronic diseases, including cancer. There is a pressing need to develop our knowledge regarding interactions of food contaminants and food components both in vitro and in vivo. Pesticides are highly undesirable food contaminants, and traumatic acid (TA) is a very beneficial food ingredient, therefore we decided to study if TA may act as a compound that delays the stimulatory effect of pesticides on breast cancer cells. To analyze the potential effects that selected herbicides (MCPA, mesotrione, bifenox and dichlobenil) may have upon cancerous cells, we conducted studies of the cytotoxicity of physiological concentrations of four pesticides and the mix of TA with tested herbicides in three different breast cancer cell lines (MCF-7, ZR-75-1 and MDA-MB-231) and one normal healthy breast cell line MCF-12A. Based on the obtained results we conclude that TA in a concentration-dependent manner might influence selected effects of the studied herbicides for particular cancer cells lines.

## 1. Introduction

In recent years, a growing body of evidence has shown that pesticides, particularly herbicides used routinely in crop production may cause detrimental effects upon human health. The main consequence of the use of pesticides is the presence of their residues in food of plant origin. Therefore, people are especially exposed to different types of plant protection products through the consumption of raw fruits and vegetables [1]. Pesticides are easily absorbed through the gastrointestinal and respiratory tract and skin. Due to their high stability and their affinity to adipose tissue they can metabolize and be stored in human organs mainly in adipose tissue. Pesticides high toxicity and extreme persistence in the environment are the reasons of special attention, which should be paid to herbicide contamination [2]. Herbicides belong to a very widely used group of pesticides because of the weeds excellent adaptation to different habitat conditions. Properly used herbicides are one of the main tools that support farmers in achieving optimal crop yields. However at the end of the 20^th^ century appeared a worldwide trend to limit the use of pesticides, especially promoted by the European Union (EU) and the United States (US). It resulted from the observed potential toxic side effects of herbicides. Pro-ecological policy promoted in the countries of the EU and the US was related to the new strategy of crop protection. According to this strategy the reduction of doses and frequency of chemical usage to the necessary minimum was defined [3]. The reduction in the use of herbicides had enormous economic and environmental consequences for agricultural development, because the evaluation of weeds in terms of their resistance to herbicides was associated with complicated and expensive procedures [4]. 

Commonly used pesticides whose chemical structure is derived from diphenyl ether (bifenox), triketone (mesotrione), aryloxyalkanoic acid (MCPA) and benzonitrile (dichlobenil) were selected for the study because of the lack of information regarding their toxicity, especially on the molecular level. The functional consequences of the possible interactions of selected pesticides with cancer and healthy cells of breast tissue have not yet been examined in details. Thus it becomes pertinent to study the molecular and physiological effects of these compounds. The other very important reason to investigate their influence on breast cancer cells is their increasing use for crop protection.

Bifenox (methyl 5-(2,4-dichlorophenoxy)-2-nitrobenzoate) belongs to the group of herbicides and it is used in the control of annual broad-leaved weeds and some grasses in numerous crops, e.g., in cereals, maize, soya beans, rice and other crops. It is present in the freshwater environment, is highly toxic to algae and exerts a specific mode of action. It causes cellular membrane damage in plant cells and an inhibition of photosynthesis. Bifenox also blocks protoporphyrinogen oxidase and participates in oxygen radical generation in the presence of light [5,6]. Currently there is no data available regarding bifenox influence on human cancer cells on the molecular level. Only a short literature data regarding its toxicity towards mammals is available. 

Mesotrione (2-(4-mesyl-2-nitrobenzoyl)cyclohexane-1,3-dione) belongs to the group of herbicides that inhibit the enzyme HPPD (4-hydroxyphenylpyruvate dioxygenase). This enzyme is necessary both in plant and animal metabolism: In carotenoid biosynthesis in plants and in tyrosine metabolic breakdown in mammals [7]. Mesotrione is classified as hazardous for the aquatic environments according to its safety data sheet. Literature data indicate that mesotrione metabolites are more toxic than a parent molecule [8]. Although mesotrione is considered to be a compound that has a very low toxicity to mammals and honeybees, there is no available data regarding its activity in human cells and its influence on human cancer cells.

Dichlobenil (2,6-dichlorobenzonitrile) is a broad-spectrum herbicide and has been widely used to control weeds from a range of genera. Although dichlobenil is persistent herbicide it is also a relatively volatile as compared to other herbicides. Therefore it can be transported via rivers and through the atmosphere [9,10,11]. In Europe dichlobenil has been banned since 2008, which is important considering the fact that its main metabolites have the capacity to reach ground water where they can give rise to transformation products that may be potentially toxic [12]. According to the literature compounds from the group of ortho-chlorobenzamide derivatives, such as dichlobenil, may react with DNA to form adducts and should be considered a potential mutagen, carcinogen and teratogen [13,14].

MCPA (4-chloro-o-tolyloxyacetic acid) belongs to the class of chlorophenoxyacetic acid compounds, which constitute an important class of commercially available pesticides. Due to its effectiveness and low costs MCPA is widely used in many types of crops, e.g., rice, tobacco, oil palm, cocoa, rubber, sugar cane and in the control of algal bloom in reservoirs [15,16]. In animals MCPA causes an inhibition of mitochondrial enzymes, which subsequently influences cell energy metabolism. It also influences the activity of enzymes that produce polyamines essential for protein synthesis and inhibits DNA synthesis. According to the literature this compound induces hepatic enzymes involved in detoxification and lipid peroxidation [17,18]. There is very little data available regarding MCPA toxicity in human cell culture, especially its influence on breast cancer development.

One of the methods of eliminating the negative effects of plant protection products is the use of substances with antioxidant activity, due to pesticides pro-oxidative mechanism of action. A little known compound with antioxidant activity and potential anticancer properties is traumatic acid (TA), which in plants has a protective, repairing and restoring role. Very important aspects are also interactions of food contaminants and food components both in vitro and in vivo. Pesticides belong to the group of highly undesirable food contaminants, and traumatic acid falls into the category of very beneficial food ingredients. Previous works from our laboratory have shown that TA acts as an antioxidant in vitro in normal human fibroblast cells and on the other hand it acts as prooxidant in breast cancer cells [19]. TA is an oxidative derivative of unsaturated fatty acids, which is synthesized in the area of plant injury either from linoleic or linolenic acid [20,21]. Selected types of lipids, especially ω-3 fatty acids, influence positively on human organs acting as inhibitors of tumor growth but on the other hand ω-6 fatty acids have a well established role in tumor development promotion [22]. Due to the strong connection between the consumption of dietary fat and the occurrence of breast cancer we decided to study if TA may act as a compound that delays the stimulatory effect of pesticides on breast cancer cells.

To analyze the potential effects that selected herbicides may have upon cancerous and non-cancerous cells, we have now conducted studies of the cytotoxicity of physiological concentrations of four pesticides in three different breast cancer cell lines and one normal breast cell line commonly utilized in breast cancer research. In our experiment we utilized MTT assay, which is one of the most popular and widely used tests to study the influence of selected compounds on relative cell viability. One of the cell lines is MCF-7, which is commonly used in the studies regarding estrogen-dependent transactivation because of its exquisite hormone sensitivity through expression of estrogen receptor, making it an ideal model for hormone response study [23]. The second cell line—ZR-75-1 belongs to the Luminal B subtype in molecular classification of breast carcinoma, which is a subtype that is usually endocrine responsive [24]. The third cell line is MDA-MB-231, which represents another mammary epithelial carcinoma that is not considered estrogen-dependent because it does not express the estrogen receptor [25]. The fourth cell line MCF-12A was developed from normal, epithelial mammary cells and it is not generally considered as estrogen-responsive. By choosing these four different cell lines as a research model in our experiments, we can compare the potentially cytotoxic or stimulatory effect of physiologically relevant concentrations of tested herbicides on both estrogen-dependent and estrogen-independent breast cancers as well as on normal, non-cancerous cells. We conducted our studies in the wide range of concentrations because although it is important to look at the effect of a large dose of studied compounds, it is even more physiologically relevant to examine the influence of concentrations of pesticides that are present in the environment. We decided to investigate the effect of herbicides that are rather widely used in crop protection in Poland. This will help in answering the question whether exposure to selected herbicides is potentially dangerous for healthy women and women diagnosed with breast cancer and if TA may possibly delay the effect of pesticides present in food.

## 2. Results

### 2.1. Effects of Tested Pesticides on Cell Viability

**Figure 1:**
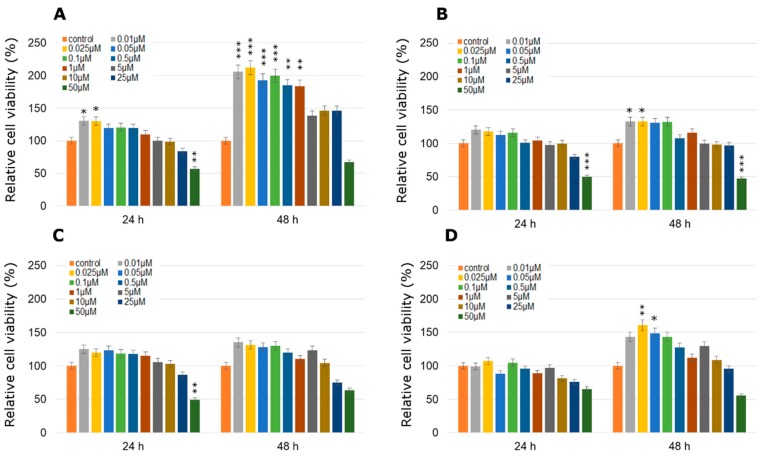
Cell viability results using MTT for MCF-7 cells exposed to graded concentrations of pesticides (**A**—MCPA, **B**—mesotrione, **C**—bifenox and **D**—dichlobenil) for 24 h and 48 h calculated as a percentage of control, untreated cells. Each value on the graph is the mean of three independent experiments and error bars show the standard error of means (SEM). * *p* < 0.05, ** *p* < 0.01 and *** *p* < 0.001 represent significant effects between treatments and control followed by a Dunnett’s test.

With single pesticide treatment at doses between 0.01 µM and 0.1 µM for 48 h, MCPA was the most stimulatory to MCF-7 cells, enhancing the cell viability in a dose dependent manner compared to control, untreated cells (Figure 1A). The concentration of 0.025 µM caused an increase in cell viability by about 112% compared to control cells. The second very active towards MCF-7 cells compound was mesotrione, especially in two concentrations: 0.01 µM and 0.025 µM after 48 h of treatment (by about 32% compared to the control in both cases) and the next one was dichlobenil in 0.025 µM concentration also after 48 h treatment (by about 60% compared to the control; Figure 1B,D). The above mentioned increases in cell viability were statistically significant. In the case of 24 h treatment with MCPA in 0.01 µM and 0.025 µM concentration an increase of 30% was observed compared to the untreated control cells. Bifenox caused the highest increase by about 35% in cell viability in 0.01 µM concentration (Figure 1C). However, treatment with high concentrations of every tested pesticide in MCF-7 cell line caused decreases in cell viability directly proportional to the increasing concentrations of pesticides. 

**Figure 2:**
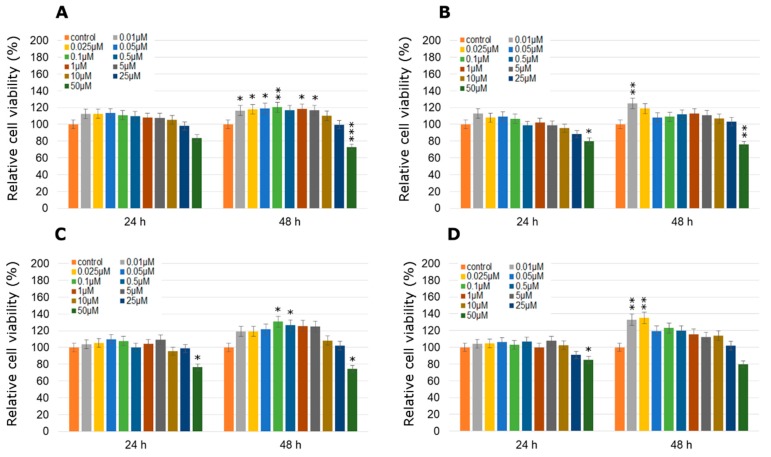
Cell viability results using MTT for MDA-MB-231 cells exposed to graded concentrations of pesticides (**A**—MCPA, **B**—mesotrione, **C**—bifenox and **D**—dichlobenil) for 24 h and 48 h calculated as a percentage of control, untreated cells. Each value on the graph is the mean of three independent experiments and error bars show the standard error of means (SEM). * *p* < 0.05, ** *p* < 0.01 and *** *p* < 0.001 represent significant effects between treatments and control followed by a Dunnett’s test.

In the case of the MDA-MB-231 cell line we did not obtain such a significant response for pesticides treatment as in the other tested cell lines (Figure 2). Statistically significant increases in cell viability were not observed with the exception of MCPA, which was the most effective in cancer cell growth stimulation. At 0.1 µM MCPA concentration after 48 h treatment an increase of about 20% as compared to the control in cell viability was observed. The other tested pesticides did not cause statistically significant increases in cell viability after 24 h of treatment. Only after 48 h of treatment in selected concentrations did they exhibit a stimulatory effect on MDA-MB-231 cell viability. 

**Figure 3:**
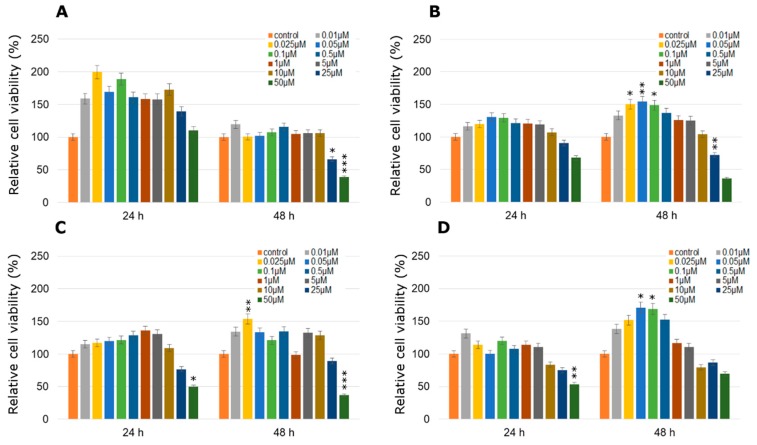
Cell viability results using MTT for ZR-75-1 cells exposed to graded concentrations of pesticides (**A**—MCPA, **B**—mesotrione, **C**—bifenox and **D**—dichlobenil) for 24 h and 48 h calculated as a percentage of control, untreated cells. Each value on the graph is the mean of three independent experiments and error bars show the standard error of means (SEM). * *p* < 0.05, ** *p* < 0.01 and *** *p* < 0.001 represent significant effects between treatments and control followed by a Dunnett’s test.

Every one of the tested herbicides significantly stimulated the proliferation of ZR-75-1 cells (Figure 3). Significantly high increases in cell viability were observed especially for the mesotrione and MCPA treatment (Figure 3A,B). MCPA caused statistically significant changes in the tested parameter right after 24 h treatment, especially in 0.025 µM (an increase by about 99%) and 0.1 µM (an increase by about 89%) concentration. On the other hand, mesotrione caused an increase in cancer cell viability by about 50% in three concentrations: 0.025 µM, 0.05 µM and 0.1 µM. Similarly bifenox and dichlobenil were more efficient in cancer cell growth stimulation after a longer treatment (48 h) in lower concentrations (0.025 µM for bifenox and 0.05 µM and 0.1 µM for dichlobenil; Figure 3C,D). In all conducted experiments the highest pesticide concentrations were cytotoxic for cancer cells. These concentrations were very high and do not occur in the environment. The lowest tested concentrations were equivalent to environmental pesticides concentration in water and food ingredients.

**Figure 4:**
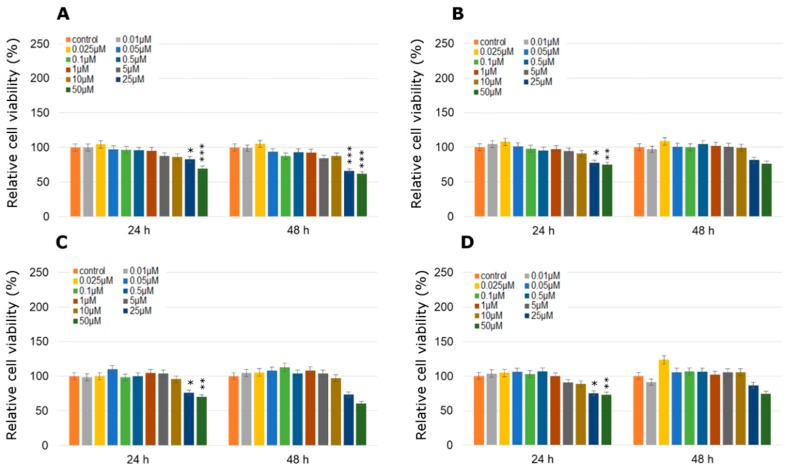
Cell viability results using MTT for MCF-12A cells exposed to graded concentrations of pesticides (**A**—MCPA, **B**—mesotrione, **C**—bifenox and **D**—dichlobenil) for 24 h and 48 h calculated as a percentage of control, untreated cells. Each value on the graph is the mean of three independent experiments and error bars show the standard error of means (SEM). * *p* < 0.05, ** *p* < 0.01 and *** *p* < 0.001 represent significant effects between treatments and control followed by a Dunnett’s test.

To examine the possible effects of physiological and environmental concentrations of different herbicides used in Poland crop production, MCF-12A mammary epithelial cells were also used. Obtained results indicated that statistically non-significant decreases in cell viability were observed (Figure 4). Only the three highest concentrations (10 µM, 25 µM and 50 µM) caused significant decreases in cell viability. It could probably be explained by the high toxicity of these concentrations, however they are rather unprecedented in the environment or food. 

### 2.2. TA Cytotoxicity 

**Figure 5:**
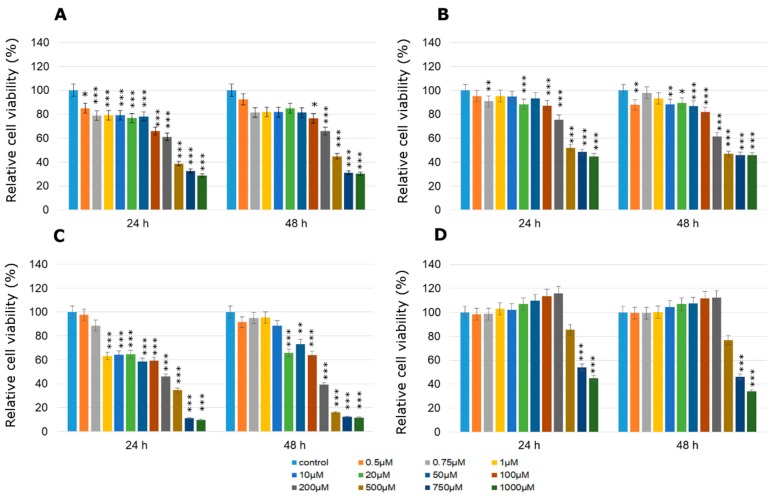
Cell viability results using MTT for MCF-7 (**A**), MDA-MB-231 (**B**), ZR-75-1 (**C**) and MCF-12A (**D**) cells exposed to graded concentrations of traumatic acid for 24 h and 48 h calculated as a percentage of control, untreated cells. Each value on the graph is the mean of three independent experiments and error bars show the standard error of means (SEM). * *p* < 0.05, ** *p* < 0.01 and *** *p* < 0.001 represent significant effects between treatments and control followed by a Dunnett’s test.

TA caused significant decreases in breast cancer cell viability in three analyzed breast cancer cell lines and increases in mammary epithelial healthy cells. Observed declines were dependent on substance dose. Statistically significant results were observed in MCF-7 cell line after 24 h treatment in every tested TA concentration (Figure 5A). Even in low concentrations, such as 0.5 µM, 0.75 µM, 1 µM and 10 µM TA was cytotoxic to MCF-7 cells causing a decrease in cell viability to approximately 80%. Longer incubation time caused also decreases in cell viability but in rather higher tested concentrations starting with 100 µM. TA was also very effective in reducing viability of ZR-75-1 cell line in both incubation times (Figure 5C). After 24 h treatment a statistically significant decline by about 40% in 1 µM concentration of TA was observed. TA concentration of 100 µM caused more than a 50% decline after 24 h and more than a 60% decline after 48 h of incubation in the ZR-75-1 cell line. The weakest response for TA treatment was given by MDA-MB-231 cell line, however it was also statistically significant (Figure 5B). In 200 µM concentration a decrease by about 25% after 24 h and approximately 40% after 48 h was observed. Higher than 200 µM concentrations induced more significant declines, however they were also toxic to normal healthy cells. In the case of the MCF-12A cell line increases were observe in both times of incubation and they amounted to 15% at the most at 200 µM of TA concentration. The concentrations of 10 µM TA and lower did not cause any changes in the relative cell viability (Figure 5D), however concentrations of 500 µM and higher were cytotoxic to MCF-12A cells and caused significant decreases in cell viability.

### 2.3. Cytotoxicity of TA Combined with Pesticides in Selected Concentrations

**Figure 6:**
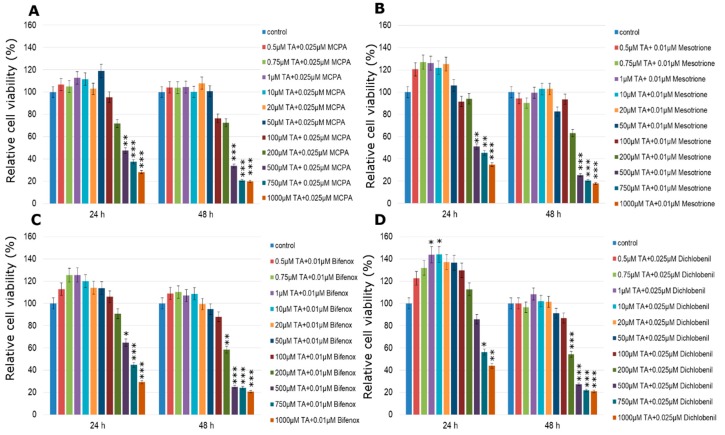
Cell viability results using MTT for MCF-7 cells exposed to chemically defined combinations of pesticides (**A**—MCPA, **B**—mesotrione, **C**—bifenox and **D**—dichlobenil) and traumatic acid for 24 h and 48 h calculated as a percentage of control, untreated cells. Each value on the graph is the mean of three independent experiments and error bars show the standard error of means (SEM). * *p* < 0.05, ** *p* < 0.01 and *** *p* < 0.001 represent significant effects between treatments and control followed by a Dunnett’s test.

**Figure 7:**
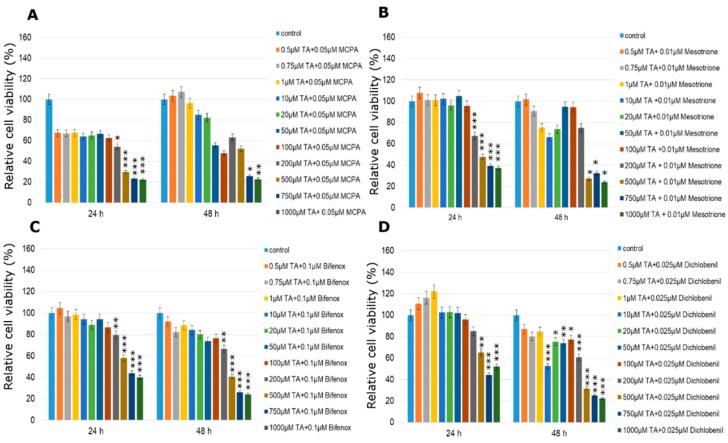
Cell viability results using MTT for MDA-MB-231 cells exposed to chemically defined combinations of pesticides (**A**—MCPA, **B**—mesotrione, **C**—bifenox and **D**—dichlobenil) and traumatic acid for 24 h and 48 h calculated as a percentage of control, untreated cells. Each value on the graph is the mean of three independent experiments and error bars show the standard error of means (SEM). * *p* < 0.05, ** *p* < 0.01 and *** *p* < 0.001 represent significant effects between treatments and control followed by a Dunnett’s test.

**Figure 8:**
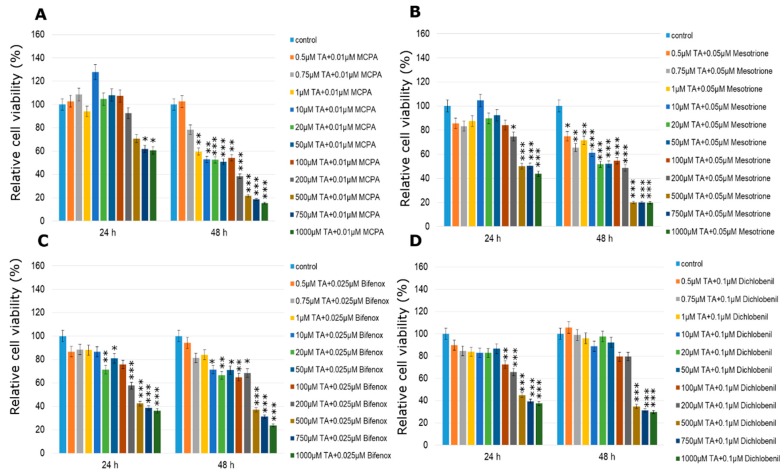
Cell viability results using MTT for ZR-75-1 cells exposed to chemically defined combinations of pesticides (**A**—MCPA, **B**—mesotrione, **C**—bifenox and **D**—dichlobenil) and traumatic acid for 24 h and 48 h calculated as a percentage of control, untreated cells. Each value on the graph is the mean of three independent experiments and error bars show the standard error of means (SEM). * *p* < 0.05, ** *p* < 0.01 and *** *p* < 0.001 represent significant effects between treatments and control followed by a Dunnett’s test.

**Figure 9:**
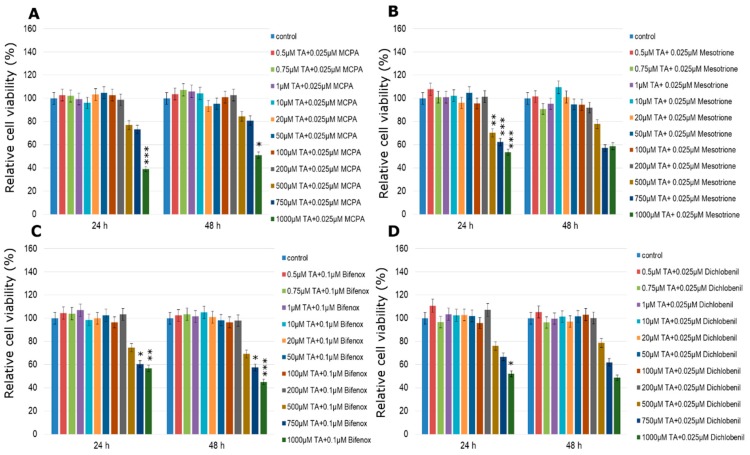
Cell viability results using MTT for MCF-12A cells exposed to chemically defined combinations of pesticides (**A**—MCPA, **B**—mesotrione, **C**—bifenox and **D**—dichlobenil) and traumatic acid for 24 h and 48 h calculated as a percentage of control, untreated cells. Each value on the graph is the mean of three independent experiments and error bars show the standard error of means (SEM). * *p* < 0.05, ** *p* < 0.01 and *** *p* < 0.001 represent significant effects between treatments and control followed by a Dunnett’s test.

One, the most stimulatory concentration of every pesticide was selected and combined with all of the tested concentrations of TA (ranged from 0.5 µM to 1000 µM). In MCF-7 cell line cytotoxicity of the studied combination of TA in different concentrations with every one of studied pesticides was significantly high only in the three highest TA concentrations (Figure 6). The only one exception was the mix of TA (500 µM) with dichlobenil after 24 h treatment (Figure 6D). In the range of lower TA concentrations, TA was not efficient enough to minimize the stimulating effect of the pesticide. On the other hand, treatment with TA MCPA-stimulated MDA-MB-231 cells caused significant declines in cell viability right after 24 h, which was statistically significant especially in the four highest concentrations (Figure 7A). Similarly the stimulating effect of mesotrione was hardly altered by the addition of TA and in cases where it occurred, it was only in the highest doses tested (200–1000 µM; *p* < 0.001) (Figure 7B). Decreases in MDA-MB-231 cell line viability were observed when cells were treated with the combination of bifenox and TA in the total range of concentrations (Figure 7C). Statistically significant effects were observed in the highest TA concentrations (*p* < 0.001; 500–1000 µM TA). The most significant effects were observed in MDA-MB-231 cell line viability treated with the combination of dichlobenil with TA (Figure 7D). After 48 h incubation declines were observed in every tested concentration. In 50 µM TA concentration a statistically significant (*p* < 0.001) decline in cell viability by about 50% was observed. As a result of using the combination of TA with MCPA in the ZR-75-1 cell line, a decrease in cell viability was noticed (Figure 8A). The most significant decline in the tested parameter was observed after 48 h of incubation with the mix of both studied compounds. Statistically significant data were obtained even for 1 µM concentration of TA (*p* < 0.01), as well as 20 µM and 50 µM of TA with an even higher probability (*p* < 0.001). Similar effects were observed under the influence of mesotrione combined with TA (Figure 8B). Statistically significant decreases in cell viability were observed in every tested TA concentration after 48 h incubation. The bifenox stimulatory effect in the ZR-75-1 cell line was also reduced by the addition of TA, especially after 48 h incubation in almost every tested concentration (Figure 8C). In the case of dichlobenil, TA was efficient in reducing pesticide-stimulated cell viability, but statistically significant results were obtained only for higher TA concentrations starting from 100 µM (*p* < 0.01) after 24 h incubation and from 500 µM after 48 h incubation (*p* < 0.001; Figure 8D). In the case of MCF-12A treated with MCPA, little variation from the control was observed, although a significant almost 5% increase in cell density was observed at 10 µM concentration of TA mixed with 0.025 µM MCPA. The treatment with 0.025 µM of mesotrione caused a significant increase in MCF-12A cell viability and also at 10 µM concentration of TA. Bifenox combined with TA caused non-significant increases and non-significant decreases in MCF-12A cells viability. In combination with dichlobenil, TA was efficient in influencing pesticide-reduced cell viability in 0.5 µM and 200 µM (Figure 9). 

**Figure 10:**
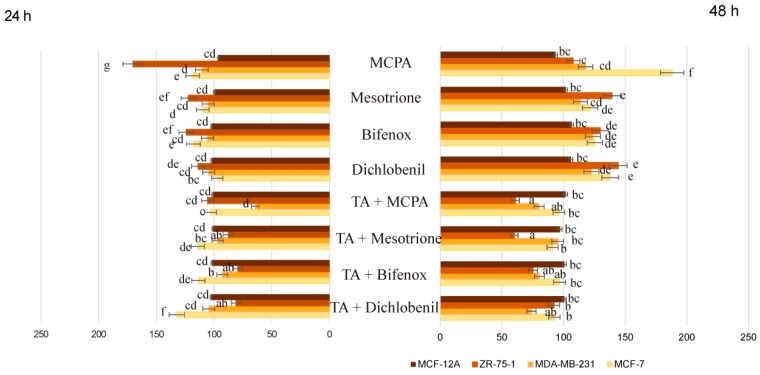
Cytotoxicity of pesticides and chemically defined combinations of pesticides and traumatic acid in four breast cell lines. Results are shown as a percentage of live cells compared to control, untreated cells set at 100 percent, with no regard to the concentration of pesticide and pesticide and TA mixture used. Mean values from three independent experiments ± SEM are shown. Different letters indicate statistical differences (*p* ≤ 0.05) between each treatment estimated by a Tukey’s test.

Summarizing, the influence of TA on the viability reduction of pesticide-treated breast cancer and non-cancerous cells is clearly presented in Figure 10. Obtained results are shown as a percentage of live cells compared to control untreated cells set at 100 percent, with no regard both to the concentration of pesticide used and the concentration of TA. The differences in cell viability in cells treated with pesticides and cells treated with pesticides combined with TA were especially easily noticeable after 48 h incubation. From this graph it could be deduced that every studied pesticide without considering its concentration had caused a significant increase in cancer cell viability compared to the control. The highest stimulation level was observed in the case of the MCF-7 cell line and MCPA, however viability of ZR-75-1 cells was also increased under the influence of mesotrione, bifenox and dichlobenil. Interestingly, co-treatment of pesticide-treated cultures with TA had a significant impact on the cell viability decreasing it to a great extent after 48 h treatment. In every tested combination the level of viable cell did not exceed 100% control untreated cells. In the case of MCF-12A cells we did not notice any significant changes in relative cell viability under the influence of tested compounds. 

The measured and expected effects of different combinations of TA with all tested pesticides treatments at the examined doses in four analyzed cell lines can be seen in Supplementary File (Supplementary Figures). It was observed that the measured effect of MCPA combined with different concentrations of TA on MCF-7 cells was significantly different than expected, especially for the 48 h treatment. A similar situation was noticed in the case of dichlobenil 48 h treatment, but a shorter time of incubation with dichlobenil combined with TA showed that the measured values were higher than expected. In MDA-MB-231 cell line significant differences between measured and expected values were observed, especially after the 48 h treatment. Only MCPA mixed with TA for 24 h revealed that measured values of relative cell viability were significantly lower than expected. Similarly in ZR-75-1 cell line significant differences between measured and expected values of relative cell viability especially after 48 h treatment were noticed. In every analyzed pesticide at 48 h treatment measured values of relative cell viability were lower than expected. Therefore the combinatory effect of analyzed pesticides and TA on three cancer cell lines in 48 h time of incubation was antagonistic in nature for all the doses tested, except in the MDA-MB-231 cell line treated with MCPA when there was an exhibition of additive effect in lower TA concentrations. On the other hand in MCF-12A cell line measured values were rather lower than expected and we observed mainly a synergistic effect of the studied compound, which needs further examination, especially regarding molecular mechanisms, which may plan an important role in analyzed compounds interactions (Table S1. Supplementary File). 

## 3. Discussion

Environmental pollution is a worldwide phenomenon, and the risks that it brings and the effects on human health are very worrying factors. The current level of environmental pollution is the result of human activities, although the benefit-risk ratio resulting from the use of plant protection products must also be equally taken into account. The obvious issue is that food and water are the basic human needs of life. On the other hand, the explosion of the human population has caused a sudden and burdensome need for large amounts of food and, consequently, an increase in food production. Therefore, it is necessary to produce better yields and protect farm yields against diseases and insects. As a result, various types of chemical preparations used as plant protection agents are increasingly used in agriculture all over the world [26]. Pesticides can be classified into many different groups according to their activity or according to their chemical structure. For example based on their target of predator or pest they can be divided into insecticides, herbicides and fungicides.

Pesticides exposure has been reported in food and human exposure studies worldwide and is directly linked to many chronic diseases and mortality [27]. It results from pesticides properties that allow them to reach easily to every organ and system in the human body including endocrine glands, reproductive, nervous, cardiovascular, respiratory, immune and renal system. It has been reported that pesticides, especially herbicides, may induce cancerogenesis in human and animals. Literature data indicate that contamination with herbicides is directly associated with a carcinogenic effect, such as lung, breast, pancreatic, brain, prostate, stomach, ovarian and kidney cancers, as well as non-Hodgkin lymphoma, and leukemia [28]. In turn, traumatic acid (TA) falls into a category of very beneficial food ingredients. Previous works from our laboratory have shown that TA acts as an antioxidant in vitro in normal human fibroblast cells and on the other hand it acts as a prooxidant in breast cancer cells. Due to the fact that there is a strong connection between the consumption of dietary fat and the occurrence of breast cancer we decided to study if TA may act as a compound that delays the stimulatory effect of pesticides on breast cancer cells.

Therefore, this study investigated the individual effect of selected pesticides, traumatic acid and the combined effect of these compounds and evaluated their interactive influence on three different breast cancer cell lines. Pesticides were selected because they are common contaminants in food and they are widely used in Poland in crop protection. Stimulatory effect on cancer cells of one of these pesticides—MCPA—was being indicated in previous studies, which prompted us to expand the research topic and extend it to other breast cancer cell lines and other herbicides commonly used in plant protection in Poland [29].

### 3.1. The Effect of Individual Pesticides on Breast Cancer and Non-Cancerous Cells

In our experiments we found that the most stimulatory pesticide was MCPA, especially in MCF-7 and ZR-75-1 cell lines at the doses tested. Such effect of MCPA on MCF-7 cells was observed previously [29]. According to Lin and Garry a significantly high level of MCF-7 cells proliferation was observed under the influence of selected herbicides [30]. Kogevinas et al. showed that among workers exposed to chlorophenoxy herbicides an excess cancer incidence was observed [31]. Whereas, Florian et al. reported that MCF-7 and MDA-MB-231 treatment with selected herbicides did not influence significantly relative cell viability, but it caused changes in the ability of cells to regulate the amount of GPR30 (a membrane-bound G protein coupled receptor, recognized as a nonclassical receptor for estrogen and a potential receptor for triazines) transcript produced when cells are exposed to an environmental ligand [32,33]. The highest concentrations of analyzed pesticides were cytotoxic to all three cell lines, however concentrations above 5 µM should be treated with caution because human beings usually are not exposed to such concentrations of pesticides during normal consumption of fruits, vegetables and crops.

Our results indicating an increase in cell viability under the influence of almost all of the tested pesticides are similar to that described by Rich et al., which demonstrated that cytotoxicity was not observed in the estrogen-dependent MCF-7 mammary epithelial carcinoma cells; rather increases in cell viability were seen for some of the compounds at select concentrations [34]. We observed that for most of the compounds examined MDA-MB-231 cells showed trends toward a very low, non-significant increases in cell viability compared to the control cells, and the only exception was the 48 h treatment with MCPA. It is in agreement with results obtained by Rich et al., who observed slight and non-significant decreases in MDA-MB-231 cells viability under the influence of some of tested compounds, but they also noticed a significant increase in the same cell line viability treated with simazine, which is a popular herbicide [34]. According to the US EPA simazine is not a carcinogenic compound, but it was shown that simazine derivatives may disrupt the hypothalamic-pituitary-gonadal axis, which, in turn, can alter the growth of mammary tissue [35,36].

Some of the concentrations of pesticides used in our experiment are outside the EPA recommended MCL levels. They are in the physiological concentration ranges for hormone tests, which is between 10 nM and 100  nM. Usually cytotoxicity studies examine μM to even mM concentrations of toxic compounds, therefore it is disturbing that such low levels of these compounds could cause changes in cell viability. The results of our research present that the lowest analyzed concentrations showed the highest activity stimulating cancer cells to proliferation.

One of the most important and striking results obtained from our research is a rather significant difference between estrogen-receptor positive and estrogen receptor negative cell lines. MCF-7 and ZR-75-1 cell lines showed a more significant response to added pesticides than the MDA-MB-231 cell line. It is possible that in the presence of an estrogen receptor some of the analyzed compounds have a stimulatory nature and therefore they enhance cell growth and proliferation. The lack of estrogen receptor on the other hand causes no observed increases in cell viability. It is in accordance with literature data indicating that selected herbicides, such as e.g., glyphosate induces breast cancer cells growth and proliferation and it occurs through the estrogen receptor [37,38]. 

For most of the compounds examined, MCF-12A cells showed trends towards a decrease in cell viability with very few exceptions. Although observed differences were minor and did not vary significantly from the control. However the mechanism of action of studied compounds in MCF-12A cells is still not known and needs further explanation and evaluation, because the lack of changes in cell proliferation does not mean that the carcinogenesis process has not already begun. 

It should be also mentioned that literature data confirmed that the amount of phenol red in current maintenance media is not high enough to stimulate estrogen responsive gene expression and therefore to stimulate an excessive cell proliferation [32]. 

### 3.2. Cytotoxic Effect of TA on Breast Cancer Cells and Non-Cancerous Cells

We currently observed a strong need to deepen our knowledge regarding interactions of food contaminants and food components both in vitro and in vivo. Pesticides are considered as highly undesirable food contaminants, and traumatic acid (TA) belongs to the category of beneficial food ingredients. Previously we revealed that TA may act as an antioxidant in vitro in normal human fibroblast cells and on the other hand as a prooxidant in breast cancer cells [19]. This is in accordance with presented results regarding the MCF-12A cell line, which represents healthy mammary epithelial cells. We observed rather non-significant increases in MCF-12A cells proliferation, however these results need further explanation in the analysis of molecular mechanisms of TA activity. Considering the fact that there is a proved connection between the consumption of dietary fat and the occurrence of breast cancer we decided to study if TA may act as a compound that is antagonistic to pesticides on breast cancer cells. However, we first tested the effect of TA itself on cell proliferation of all three analyzed cell lines. The fastest and the strongest response we observed in MCF-7 and ZR-75-1 cell lines. In MDA-MB-231 cells we noticed low decreases in relative cell viability and rather after 48 h treatment. Due to the lack of information on the impact of TA on tumor and normal cells, we can only refer to the mechanism of action in the activity of unsaturated fatty acids and their derivatives. An example may be DHA (docosahexaenoic acid), which belongs to ω-3 polyunsaturated fatty acids. According to the literature plant derived unsaturated fatty acids are proved to influence cancerogenesis [39]. They have antiproliferative and proapoptotic properties, especially ω-3 fatty acids that inhibit tumor growth. Anticancer effect is particularly well established in the case of DHA, which inhibits breast cancer cell growth, increases apoptosis and reduces cell invasiveness potential [40,41,42]. DHA also influences metalloproteinases activity and therefore it slows down breast tumor progression and inhibits metastasis. On the other hand, ω-6 fatty acids participate in tumor promotion, what has been demonstrated for arachidonic acid (AA) [22,43]. Our results are in accordance with literature data regarding DHA influence on MDA-MB-231 cells. A study conducted by Pizato et al. indicated that DHA caused a decrease in MDA-MB-231 cell viability at 200 μM [44]. We also observed that TA deceased MDA-MB-231 cells viability significantly starting from 100 µM at 24 h. Results obtained by Corsetto et al. also revealed that PUFAs (polyunsaturated fatty acids) induce cell apoptosis and cause a reduction of cell viability in two lines of human breast cancer cells characterized by a different expression of ER (estrogen receptor) and EGFR (epidermal growth factor receptor) receptors—MDA-MB-231 (ER-negative) and MCF-7 (ER-positive) [45]. The TA-induced decrease in breast cancer cells viability occurred faster in estrogen-dependent cell lines: MCF-7 and ZR-75-1. In MCF-7 cells first response for TA treatment as a decrease in relative cell viability was observed right after 24 h treatment even at the lowest concentrations of TA—0.5 µM. In ZR-75-1 cells 1 µM TA concentration caused a significant decrease at 24 h incubation. These results are confirmed by other studies that have demonstrated the cytotoxicity of DHA in breast cancer cells [41,46].

### 3.3. Effects of Combined Doses of Pesticides and TA on Breast Cancer Cell and Non-Cancerous Cells

Few studies have reported the effect of exposure of combination of pesticides and plant-derived compounds to different cell lines, but none has reported cytotoxic and interactive effects of TA mixed with herbicides on three different breast cancer cell lines and one cell line derived from healthy breast tissue. In our previous study, we have evaluated the mechanism of TA-induced cytotoxicity, apoptosis and oxidative stress in MCF-7 cell line and the activity of TA in normal, non-cancerous cells—fibroblasts. We found that in cancer cells TA acts as a prooxidant and in normal cell as an antioxidant [19]. We also conducted a preliminary study regarding selected herbicides activity in MCF-7 cells and possible mechanisms of their action in cancer cells [29]. Due to promising results that we obtained, we decided to combine these two types of compounds and verify if TA may delay the negative effects of pesticides activity and inhibit cancer cells proliferation. The addition of TA to herbicide-stimulated MCF-7 cells caused decreases in cell viability only in the highest concentrations, such as 500 µM, 750 µM and 1000 µM. These concentrations are impossible to apply in the human diet because they are cytotoxic for normal healthy cells and tissues. The stimulation of MCF-7 proliferation by pesticides is so intensive that TA treatment is not able to delay the stimulatory effect of herbicides. On the other hand, ZR-75-1 cells were significantly more susceptible for TA activity against pesticides, which was observed especially in the case of mesotrione. Every tested TA concentration of cells previously incubated with mesotrione during 48 h caused statistically significant decreases in relative cell viability. We observed a similar response in cells treated with MCPA and bifenox. 

Results obtained for MDA-MB-231 cells revealed that the highest concentrations of TA caused statistically significant decreases in herbicide-treated cells viability. Lower TA concentrations, which are possible to apply in human diet, unfortunately did not exert an expected effect.

In general, chemoprevention is known as a use of mainly natural substances, plant-derived, to slow or inhibit the negative effect of environmental contaminants, such as pesticides. Chemoprevention is mainly considered regarding the carcinogenesis process. Phytochemicals present in different plants exert a beneficial effect in cancer prevention. They can be divided according to their chemical structure into a few groups: Terpenes, phenols, sulphides, organic acids and other macromolecules [47]. Considering its chemical formula TA belongs to the group of organic acids. According to the literature data an addition of unsaturated fatty acid into diet plays an important role in amelioration of the pesticides-induced damage [48,49,50]. A protective effect of fatty acids against pesticide-induced damage was observed mainly in animal experimental model [51,52,53]. It is an undeniable fact that more research should be carried out in biological systems based on human experiments. Unfortunately, human experiments are not possible, therefore other methodologies must be used interchangeably, for example animal studies or experiments based on human cell lines, in order to mimic the reaction in human tissues as much as possible. In vitro studies regarding chemoprotective activity of fatty acids were also conducted [50,54]. Our results are in accordance with literature data indicating a decrease in pesticide-stimulated cell proliferation under the influence of fatty acid. 

A combinatory effect of pesticides and TA were calculated and estimated according to Weber et al. [55]. The measured and expected combinatory effects of TA and pesticides mixtures are presented in the Supplementary File. Combinations of TA with different pesticides in three different cell lines were generally antagonistic at 48 h of incubation. Only a shorter treatment time revealed additive and synergistic effects in a variety of TA concentrations. Obtained results indicated that longer TA treatment may delay the negative effects of pesticide influence.

**Figure 11:**
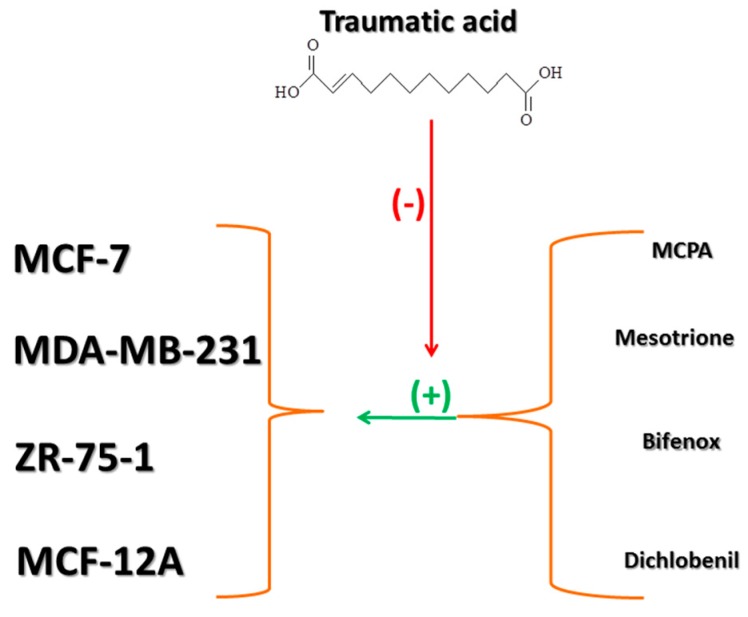
The summary of the interactions of traumatic acid (TA) and analyzed herbicides in studied cell lines.

Summarizing, in vitro exposure to different herbicides could enhance breast cancer cells proliferation leading to the possible tumor growth and development and TA could attenuate pesticide-induced cells stimulation (Figure 11). Almost every one of the tested cell lines exhibited increases in relative cell viability on different levels, except for the normal healthy cell line in which we observed a variety of effects, but was still statistically non-significant. TA activity was not cytotoxic regarding healthy cells in lower concentrations and we may presume that it delays the effect of pesticides. The three highest concentrations of TA were toxic to every tested cell line, but usually plant-derived compounds concentrations of 500 µM and higher were toxic and impossible to use in clinical studies. These observations are very interesting but they are a preliminary study and need further investigation, especially regarding mechanisms involved in TA and pesticide activity. They open a novel perspective in cancer chemoprevention by using plant-derived substances.

## 4. Materials and Methods

### 4.1. Reagents 

Dulbecco’s modified Eagle’s medium (DMEM), containing glucose at 4.5 mg/mL (25 mM), Leibovitz’s L-15 Medium, penicillin, streptomycin, trypsin–EDTA, FBS and PBS (without Ca and Mg) were provided by Gibco (San Diego, CA, USA), RPMI-1640 medium was obtained from ATCC (Manassas, VA, USA) and the MTT reagent was purchased from Sigma-Aldrich (Saint Louis, MO, USA). Traumatic acid (TA) was provided by Cayman Chemical Company (1180 East Ellsworth Road, Ann Arbor, MI, USA); purity ≥98%; formal name: 2E-dodecenedioic acid; CAS number: 6402-36-4; formulation: A crystalline solid. DMEM/F12A medium was obtained from Gibco (San Diego, CA, USA), horse serum, cholera toxin were obtained from Sigma Aldrich (Saint Louis, Missouri, USA). HEGF (human epidermal growth factor) was obtained from Roche (Roche Applied Science, Mannheim, Germany). 

### 4.2. Cell Culture

The effect of TA, pesticides and TA-pesticides was examined in three different breast cancer cell lines and one normal healthy breast cell line MCF-12A, which were obtained from American Type Culture Collection (ATCC, Manassas, VA, USA). MCF-7 cells were maintained in DMEM (Gibco, San Diego, CA, USA) supplemented with 10% FBS (Gibco, San Diego, CA, USA ), penicillin (100 U/mL) and streptomycin (100 μg/mL) at 37 °C in a humidified atmosphere of 5% CO_2_ in air. MDA-MB-231 cells were maintained in Leibovitz’s L-15 Medium supplemented with 10% FBS (Gibco, San Diego, CA, USA), penicillin (100 U/mL) and streptomycin (100 μg/mL) at 37 °C in a humidified atmosphere. ZR-75-1 cells were maintained in RPMI-1640 Medium supplemented with 10% FBS (Gibco, San Diego, CA, USA ), penicillin (100 U/mL) and streptomycin (100 μg/mL) at 37 °C in a humidified atmosphere of 5% CO_2_ in air. MCF-12A cells were maintained in DMEM/F12 media that was supplemented with 5% horse serum, 0.1 µg/mL cholera toxin, 40 ng/mL epidermal growth factor and penicillin (100 U/mL) and streptomycin (100 µg/mL) at 37 °C in a humidified atmosphere of 5% CO_2_ in air.

The cells viability was estimated at TA concentrations of 0.5 µM, 0.75 µM, 1 µM, 10 µM, 20 µM, 50 µM, 100 µM, 200 µM, 500 µM, 750 µM and 1000 µM; pesticide concentrations of 0.01 µM, 0.025 µM, 0.05 µM, 0.1 µM, 0.5 µM, 1 µM, 5 µM, 10 µM, 25 µM and 50 µM. 

Concentrations of the test subjects needed to analyze the cytotoxicity of traumatic acid mixtures with pesticides were selected based on the results of the MTT assay for TA and individual pesticides. For each pesticide, the concentration at which the greatest stimulation of proliferation was observed was selected. The experimental model containing concentrations of pesticides and TA in mixtures is presented in the Table 1. 

### 4.3. Chemical Treatment of Cells

TA was stored in a refrigerator at temperature 4 °C. TA was dissolved in DMSO. The compound was added to the cultured cells for a final concentration in the range of 0.5 µM to 1000 µM. Pesticides were stored in a refrigerator at temperature 4 °C. The compounds were added to the culture cells for a final concentration in the range of 0.01 µM to 50 µM. TA and pesticides were both added to the cultured cells in selected concentrations. The control cells were incubated without the test compounds.

### 4.4. Pesticides, TA and TA-Pesticides Cytotoxicity

TA, pesticides and TA-pesticides cytotoxicity were measured according to the method of Carmichael using 3-(4,5-dimethylthiazol-2-yl)-2,5-diphenyltetrazolium bromide (MTT) [56]. Breast cancer cells of three cell lines and one normal, healthy breast cell line were seeded in a 96-well plate at a density of 2 × 10^4^ cells/well. Cells cultured for 24 h and 48 h were treated with: 1^st^ - TA in the concentration range from 0.5 µM to 1000 µM; 2nd - pesticides in the concentration range from 0.01 µM to 50 µM; 3rd - TA combined with pesticides: TA in the concentration range from 0.5 µM to 1000 µM combined with pesticides in the selected, above described concentrations. After 24 h and 48 h, cells were washed three times with PBS and subsequently incubated with 10 µL of MTT solution (5 mg/mL in PBS) for 2 h at 37 °C in 5% CO_2_ in an incubator. The MDA-MB-231 cell line was maintained without CO_2_. Subsequently, 100 µL of DMSO was added and cells were incubated in the dark for the next 2 h. The absorbance was measured at 570 nm in a microplate plate reader GloMax^®^-Multi Microplate Multimode Reader (Promega Corporation, Madison, WI, USA). The viability of breast cancer cells and normal cells was calculated as a percentage of control cells, incubated without tested compound. All the experiments were done in triplicates. 

### 4.5. Statistical Analysis of Data

Each treatment was done in triplicates in four independent experiments and all data are given as mean values ± SEM (standard error of means). Differences between treatments and untreated control cells were analyzed by a one-way ANOVA, followed by a Dunnett’s procedure for multiple comparisons. Significant effects are represented by *p* ≤ 0.05(*), *p* ≤ 0.01 (**) and *p* ≤ 0.001(***). For the analysis of differences between each treatment (three cell lines, four pesticides and TA-pesticide mixes) one-way analysis of variance (ANOVA) followed by a Tukey’s test was applied and significance was considered when *p* ≤ 0.05.

### 4.6. Calculation of Expected Cell Viability

The expected cell viability of tested mixtures of compounds was calculated by the addition of the mean viability after exposure to traumatic acid to the mean viability after exposure to every tested pesticide. Interactions between studied compounds were calculated according to Clarke et al. [57]. The model is as follows:

Mean viability of binary mixtures (expected in % of substance 1 substance 2) = mean viability (substance 1 in %) + mean viability (substance 2 in %) − 100%.

### 4.7. Calculation of Standard Error of the Mean of Measured and Expected Values

The expected standard error of means (SEM) was calculate according to the equation:

SEM_expected_ = [(SEM_substance1_)^2^ + (SEM_substance2_)^2^]^1/2^

In the next step, the obtained measured values were compared with expected (calculated) values. If the measured values are not significantly higher or lower than expected, the effect of the two substances are additive. When the measured value is significantly higher than expected, the effect of the two substances is synergistic. If the measured effect is significantly lower than the expected value, the effect of the two substances is antagonistic.

The significance of difference was calculated using an unpaired *t*-test according to Weber et al. [55] and the effects of the mixture of the substances are shown in the table.

## 5. Conclusions 

Obtained results described in the presented paper underline the importance of nutritional factors such as TA, on health maintenance and on the prevention of many diseases, which result from long-term pesticides exposition. The most significant effect was obtained for estrogen-dependent cell lines, however almost no effect was noticed in the case of mammary epithelial healthy cells. Taken together, this preliminary study has demonstrated that TA may inhibit herbicides induced breast cancer cells proliferation depending on the type of breast cancer and therefore TA in a concentration-dependent manner might influence selected effects of the studied herbicides for particular cancer cells lines. The above presented results could be the first step in the analysis of herbicide influence on breast cancer and possible TA activity against them. However additional studies are necessary to explain mechanisms through which TA mitigates pesticides that facilitate cancer cell proliferation.

## Figures and Tables

**Table 1:** Concentrations of traumatic acid (TA) and pesticides used in the study.

	TA:MCPA										
MDA-MB-231	0.5:0.05	0.75:0.05	1:0.05	10:0.05	20:0.05	50:0.05	100:0.05	200:0.05	500:0.05	750:0.05	1000:0.05
MCF-7	0.5:0.025	0.75:0.025	1:0.025	10:0.025	20:0.025	50:0.025	100:0.025	200:0.025	500:0.025	750:0.025	1000:0.025
ZR-75-1	0.5:0.01	0.75:0.01	1:0.01	10:0.01	20:0.01	50:0.01	100:0.01	200:0.01	500:0.01	750:0.01	1000:0.01
MCF-12A	0.5:0.025	0.75:0.025	1:0.025	10:0.025	20:0.025	50:0.025	100:0.025	200:0.025	500:0.025	750:0.025	1000:0.025
	TA:Mesotrione										
MDA-MB-231	0.5:0.01	0.75:0.01	1:0.01	10:0.01	20:0.01	50:0.01	100:0.01	200:0.01	500:0.01	750:0.01	1000:0.01
MCF-7	0.5:0.01	0.75:0.01	1:0.01	10:0.01	20:0.01	50:0.01	100:0.01	200:0.01	500:0.01	750:0.01	1000:0.01
ZR-75-1	0.5:0.05	0.75:0.05	1:0.05	10:0.05	20:0.05	50:0.05	100:0.05	200:0.05	500:0.05	750:0.05	1000:0.05
MCF-12A	0.5:0.025	0.75:0.025	1:0.025	10:0.025	20:0.025	50:0.025	100:0.025	200:0.025	500:0.025	750:0.025	1000:0.025
	TA:Bifenox										
MDA-MB-231	0.5:0.1	0.75:0.1	1:0.1	10:0.1	20:0.1	50:0.1	100:0.1	200:0.1	500:0.1	750:0.1	1000:0.1
MCF-7	0.5:0.01	0.75:0.01	1:0.01	10:0.01	20:0.01	50:0.01	100:0.01	200:0.01	500:0.01	750:0.01	1000:0.01
ZR-75-1	0.5:0.025	0.75:0.025	1:0.025	10:0.025	20:0.025	50:0.025	100:0.025	200:0.025	500:0.025	750:0.025	1000:0.025
MCF-12A	0.5:0.1	0.75:0.1	1:0.1	10:0.1	20:0.1	50:0.1	100:0.1	200:0.1	500:0.1	750:0.1	1000:0.1
	TA:Dichlobenil										
MDA-MB-231	0.5:0.025	0.75:0.025	1:0.025	10:0.025	20:0.025	50:0.025	100:0.025	200:0.025	500:0.025	750:0.025	1000:0.025
MCF-7	0.5:0.025	0.75:0.025	1:0.025	10:0.025	20:0.025	50:0.025	100:0.025	200:0.025	500:0.025	750:0.025	1000:0.025
ZR-75-1	0.5:0.1	0.75:0.1	1:0.1	10:0.1	20:0.1	50:0.1	100:0.1	200:0.1	500:0.1	750:0.1	1000:0.1
MCF-12A	0.5:0.025	0.75:0.025	1:0.025	10:0.025	20:0.025	50:0.025	100:0.025	200:0.025	500:0.025	750:0.025	1000:0.025

## Supplementary Materials

The following are available online. The measured and expected additive combinatory effects of the different binary traumatic acid and selected pesticides treatments at the examined doses can be seen in supplementary figures S1–S4 and in Table S1. The overview of how the expected and measured combinatory effects of the compounds mixtures were calculated can be seen in section Materials and methods.
