# Supplementary material for: Toxicological Effects of Traumatic Acid and Selected Herbicides on Human Breast Cancer Cells: In Vitro Cytotoxicity Assessment of Analyzed Compounds
Source: Molecules. 2019 May 2;24(9):1710. doi: 10.3390/molecules24091710 (PMC6539929; doi:10.3390/molecules24091710)
Supplement: Supplementary file 1 [file molecules-24-01710-s001.pdf]

molecules

MDPI

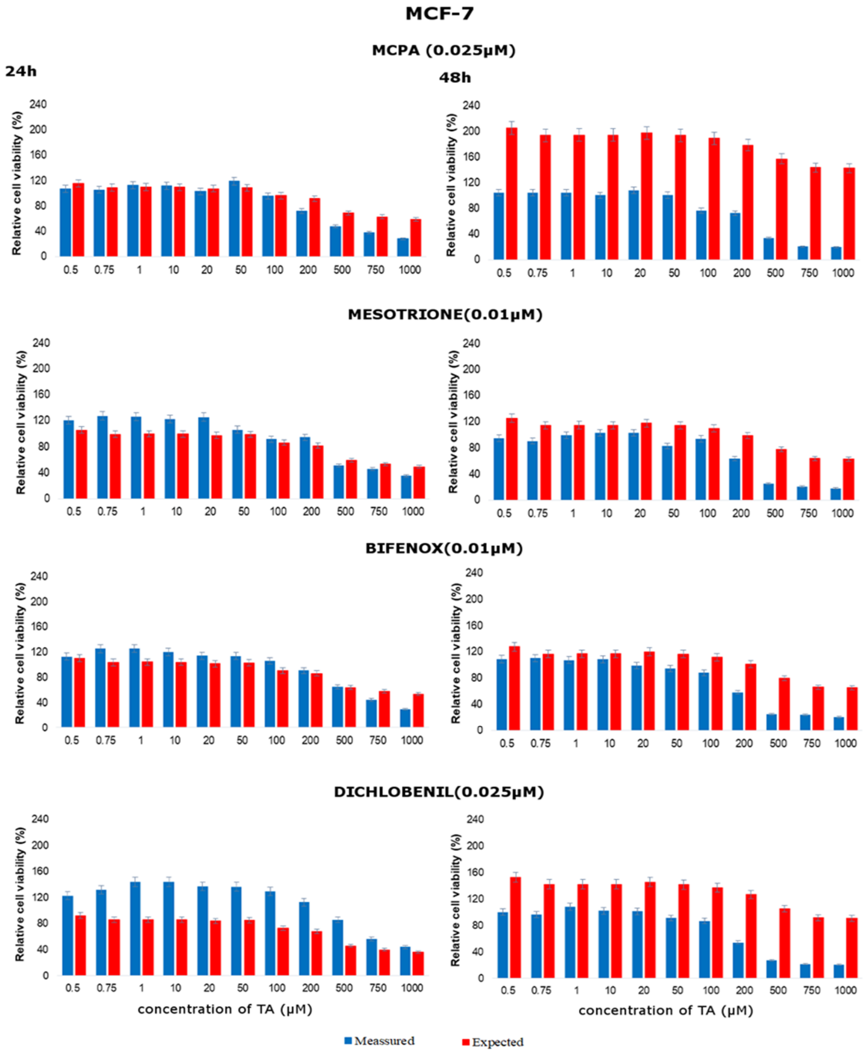

Figure 1. Graph showing the measured and expected MCF-7 cell viability of the combinations of traumatic acid and tested pesticides.

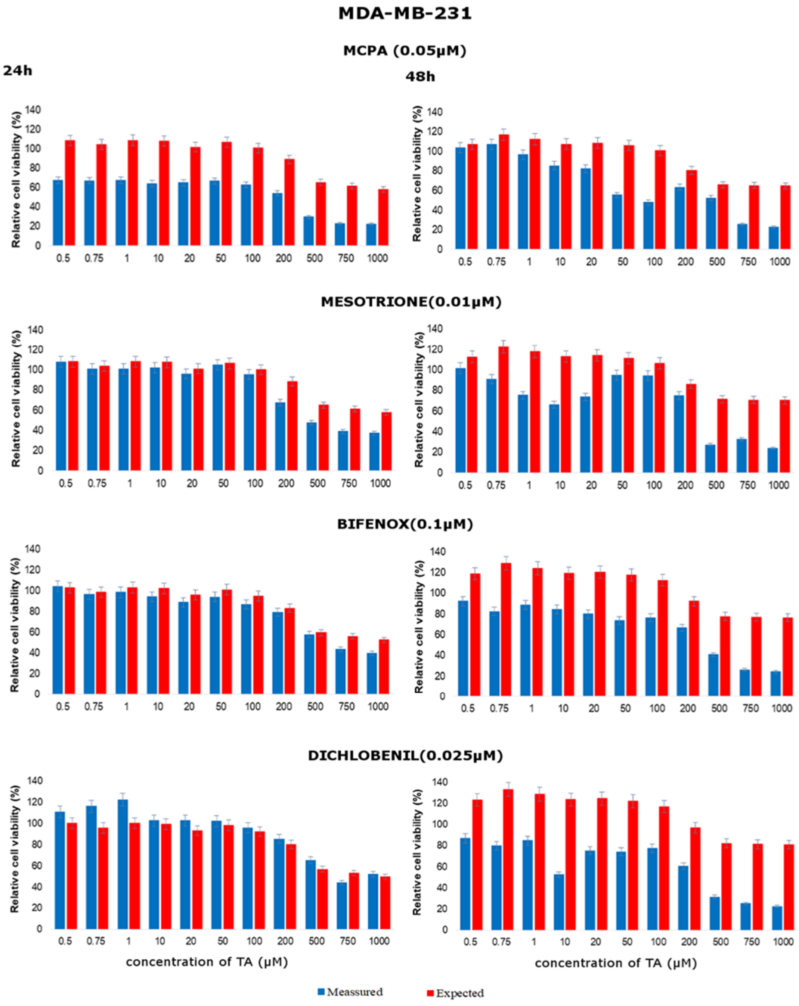

Figure 2. Graph showing the measured and expected MDA-MB-231 cell viability of the combinations of traumatic acid and tested pesticides.

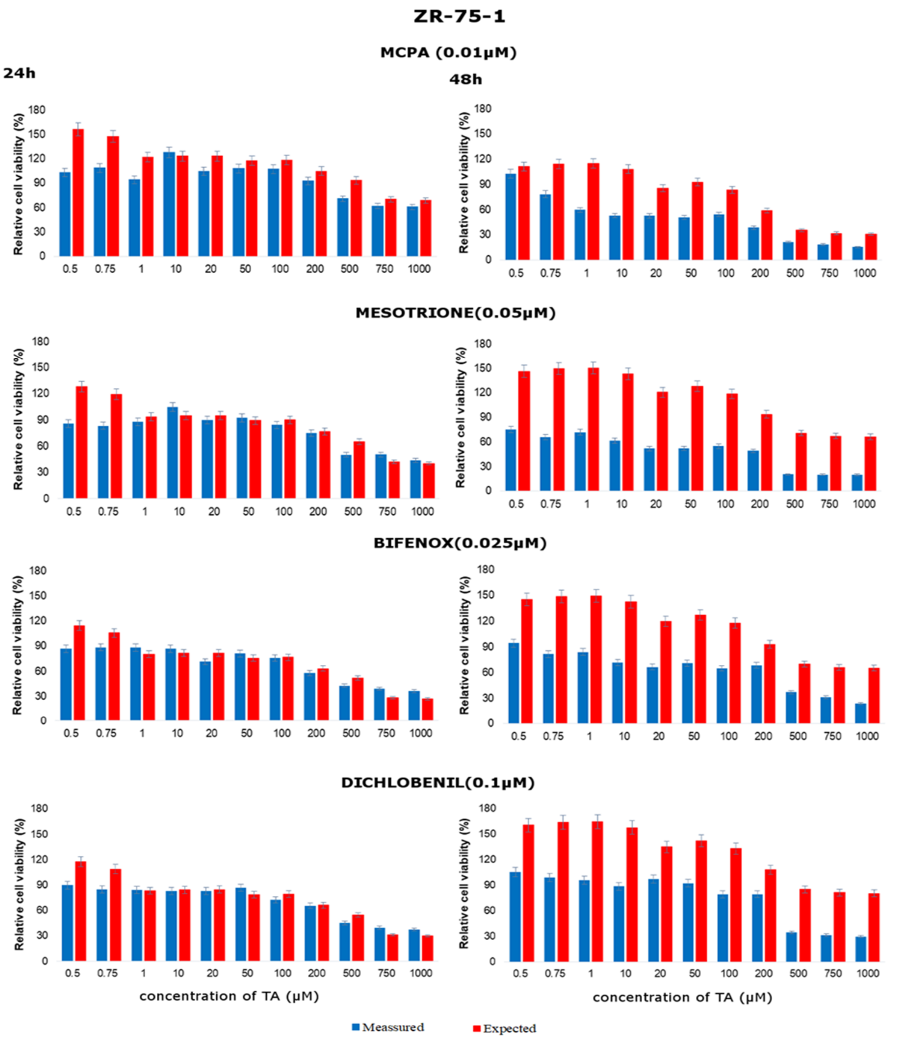

Figure 3. Graph showing the measured and expected ZR-75-1 cell viability of the combinations of traumatic acid and tested pesticides.

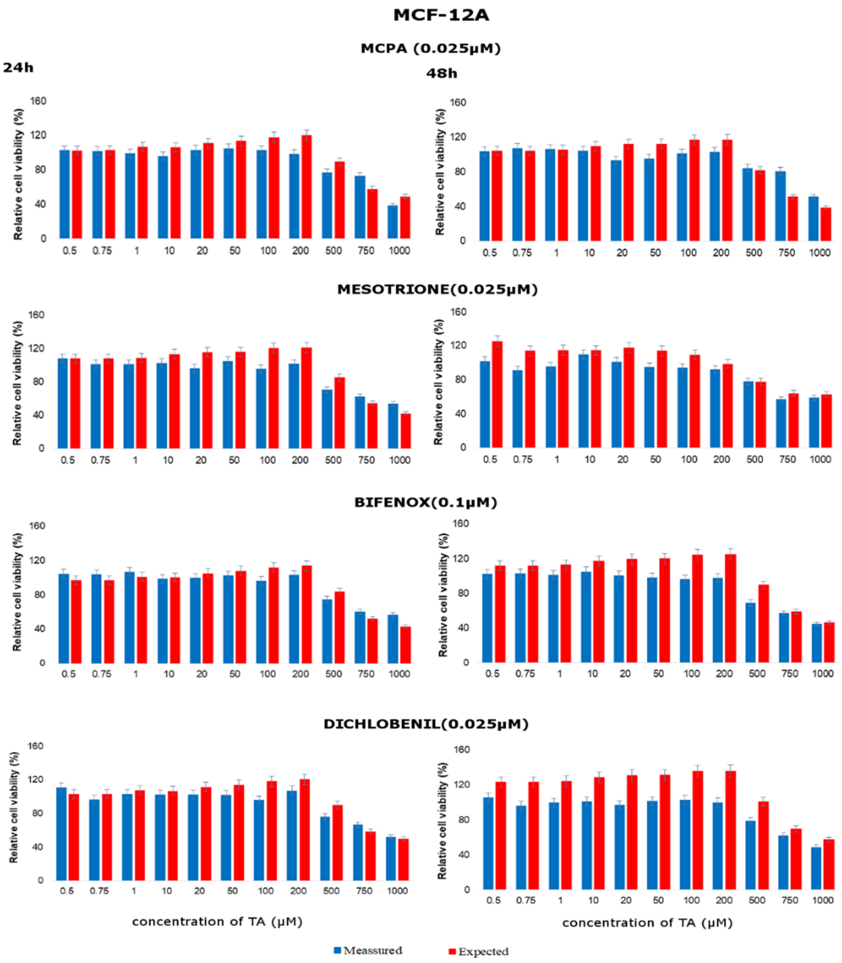

Figure 4. Graph showing the measured and expected MCF-12A cell viability of the combinations of traumatic acid and tested pesticides.

Table 1. Interactive effects of combinations of TA with selected herbicides in analyzed concentrations (antagonistic—measured cell viability values were significantly below expected values; synergistic—measured cell viability values were significantly above expected values; additive—measured cell viability values were not significantly below or above expected values; TA—traumatic acid).

dose combination (μM)		TA+MCPA		dose combination (μM)	TA+Mesotrione		dose combination (μM)	TA+Bifenox		dose combination (μM)	TA+Dichlobenil	
		24h	48h		24h	48h		24h	48h		24h	48h
MCF-7	0.5:0.025	antagonistic	antagonistic	0.5:0.01	synergistic	antagonistic	0.5:0.01	additive	antagonistic	0.5:0.025	synergistic	antagonistic
	0.75:0.025	additive	antagonistic	0.75:0.01	synergistic	antagonistic	0.75:0.01	synergistic	antagonistic	0.75:0.025	synergistic	antagonistic
	1:0.025	additive	antagonistic	1:0.01	synergistic	antagonistic	1:0.01	synergistic	antagonistic	1:0.025	synergistic	antagonistic
	10:0.025	additive	antagonistic	10:0.01	synergistic	antagonistic	10:0.01	synergistic	antagonistic	10:0.025	synergistic	antagonistic
	20:0.025	additive	antagonistic	20:0.01	synergistic	antagonistic	20:0.01	synergistic	antagonistic	20:0.025	synergistic	antagonistic
	50:0.025	synergistic	antagonistic	50:0.01	synergistic	antagonistic	50:0.01	synergistic	antagonistic	50:0.025	synergistic	antagonistic
	100:0.025	additive	antagonistic	100:0.01	synergistic	antagonistic	100:0.01	synergistic	antagonistic	100:0.025	synergistic	antagonistic
	200:0.025	antagonistic	antagonistic	200:0.01	synergistic	antagonistic	200:0.01	synergistic	antagonistic	200:0.025	synergistic	antagonistic
	500:0.025	antagonistic	antagonistic	500:0.01	antagonistic	antagonistic	500:0.01	additive	antagonistic	500:0.025	synergistic	antagonistic
	750:0.025	antagonistic	antagonistic	750:0.01	antagonistic	antagonistic	750:0.01	antagonistic	antagonistic	750:0.025	synergistic	antagonistic
	1000:0.025	antagonistic	antagonistic	1000:0.01	antagonistic	antagonistic	1000:0.01	antagonistic	antagonistic	1000:0.025	synergistic	antagonistic
MDA-MB-231	0.5:0.05	antagonistic	additive	0.5:0.01	additive	antagonistic	0.5:0.1	additive	antagonistic	0.5:0.025	synergistic	antagonistic
	0.75:0.05	antagonistic	antagonistic	0.75:0.01	additive	antagonistic	0.75:0.1	additive	antagonistic	0.75:0.025	synergistic	antagonistic
	1:0.05	antagonistic	antagonistic	1:0.01	additive	antagonistic	1:0.1	additive	antagonistic	1:0.025	synergistic	antagonistic
	10:0.05	antagonistic	antagonistic	10:0.01	additive	antagonistic	10:0.1	additive	antagonistic	10:0.025	additive	antagonistic
	20:0.05	antagonistic	antagonistic	20:0.01	additive	antagonistic	20:0.1	additive	antagonistic	20:0.025	synergistic	antagonistic
	50:0.05	antagonistic	antagonistic	50:0.01	additive	antagonistic	50:0.1	additive	antagonistic	50:0.025	additive	antagonistic
	100:0.05	antagonistic	antagonistic	100:0.01	additive	antagonistic	100:0.1	additive	antagonistic	100:0.025	additive	antagonistic
	200:0.05	antagonistic	antagonistic	200:0.01	antagonistic	antagonistic	200:0.1	additive	antagonistic	200:0.025	additive	antagonistic
	500:0.05	antagonistic	antagonistic	500:0.01	antagonistic	antagonistic	500:0.1	additive	antagonistic	500:0.025	synergistic	antagonistic
	750:0.05	antagonistic	antagonistic	750:0.01	antagonistic	antagonistic	750:0.1	antagonistic	antagonistic	750:0.025	antagonistic	antagonistic
	1000:0.05	antagonistic	antagonistic	1000:0.01	antagonistic	antagonistic	1000:0.1	antagonistic	antagonistic	1000:0.025	additive	antagonistic
ZR-75-1	0.5:0.01	antagonistic	antagonistic	0.5:0.05	antagonistic	antagonistic	0.5:0.025	antagonistic	antagonistic	0.5:0.1	antagonistic	antagonistic
	0.75:0.01	antagonistic	antagonistic	0.75:0.05	antagonistic	antagonistic	0.75:0.025	antagonistic	antagonistic	0.75:0.1	antagonistic	antagonistic
	1:0.01	antagonistic	antagonistic	1:0.05	additive	antagonistic	1:0.025	synergistic	antagonistic	1:0.1	additive	antagonistic
	10:0.01	synergistic	antagonistic	10:0.05	synergistic	antagonistic	10:0.025	additive	antagonistic	10:0.1	additive	antagonistic
	20:0.01	antagonistic	antagonistic	20:0.05	additive	antagonistic	20:0.025	antagonistic	antagonistic	20:0.1	additive	antagonistic
	50:0.01	antagonistic	antagonistic	50:0.05	additive	antagonistic	50:0.025	additive	antagonistic	50:0.1	synergistic	antagonistic
	100:0.01	antagonistic	antagonistic	100:0.05	additive	antagonistic	100:0.025	additive	antagonistic	100:0.1	antagonistic	antagonistic
	200:0.01	antagonistic	antagonistic	200:0.05	additive	antagonistic	200:0.025	additive	antagonistic	200:0.1	additive	antagonistic
	500:0.01	antagonistic	antagonistic	500:0.05	additive	antagonistic	500:0.025	antagonistic	antagonistic	500:0.1	antagonistic	antagonistic
	750:0.01	antagonistic	antagonistic	750:0.05	additive	antagonistic	750:0.025	synergistic	antagonistic	750:0.1	synergistic	antagonistic
	1000:0.01	antagonistic	antagonistic	1000:0.05	additive	antagonistic	1000:0.025	synergistic	antagonistic	1000:0.1	synergistic	antagonistic
MCF-12A	0.5:0.025	additive	additive	0.5:0.025	additive	synergistic	0.5:0.1	antagonistic	synergistic	0.5:0.025	antagonistic	synergistic
	0.75:0.025	additive	additive	0.75:0.025	synergistic	synergistic	0.75:0.1	antagonistic	synergistic	0.75:0.025	synergistic	synergistic
	1:0.025	synergistic	additive	1:0.025	synergistic	synergistic	1:0.1	antagonistic	synergistic	1:0.025	synergistic	synergistic
	10:0.025	synergistic	synergistic	10:0.025	synergistic	synergistic	10:0.1	additive	synergistic	10:0.025	synergistic	synergistic
	20:0.025	synergistic	synergistic	20:0.025	synergistic	synergistic	20:0.1	synergistic	synergistic	20:0.025	synergistic	synergistic
	50:0.025	synergistic	synergistic	50:0.025	synergistic	synergistic	50:0.1	synergistic	synergistic	50:0.025	synergistic	synergistic
	100:0.025	synergistic	synergistic	100:0.025	synergistic	synergistic	100:0.1	synergistic	synergistic	100:0.025	synergistic	synergistic
	200:0.025	synergistic	synergistic	200:0.025	synergistic	synergistic	200:0.1	synergistic	synergistic	200:0.025	synergistic	synergistic
	500:0.025	synergistic	additive	500:0.025	synergistic	additive	500:0.1	synergistic	synergistic	500:0.025	synergistic	synergistic
	750:0.025	antagonistic	antagonistic	750:0.025	antagonistic	synergistic	750:0.1	antagonistic	additive	750:0.025	antagonistic	synergistic
	1000:0.025	synergistic	antagonistic	1000:0.025	antagonistic	synergistic	1000:0.1	antagonistic	additive	1000:0.025	additive	synergistic
